# Supplementary material for: Properdin Is a Modulator of Tumour Immunity in a Syngeneic Mouse Melanoma Model
Source: Medicina (Kaunas). 2021 Jan 21;57(2):85. doi: 10.3390/medicina57020085 (PMC7909514; doi:10.3390/medicina57020085)
Supplement: Supplementary file 1 [file medicina-57-00085-s001.pdf]

Supplementary results

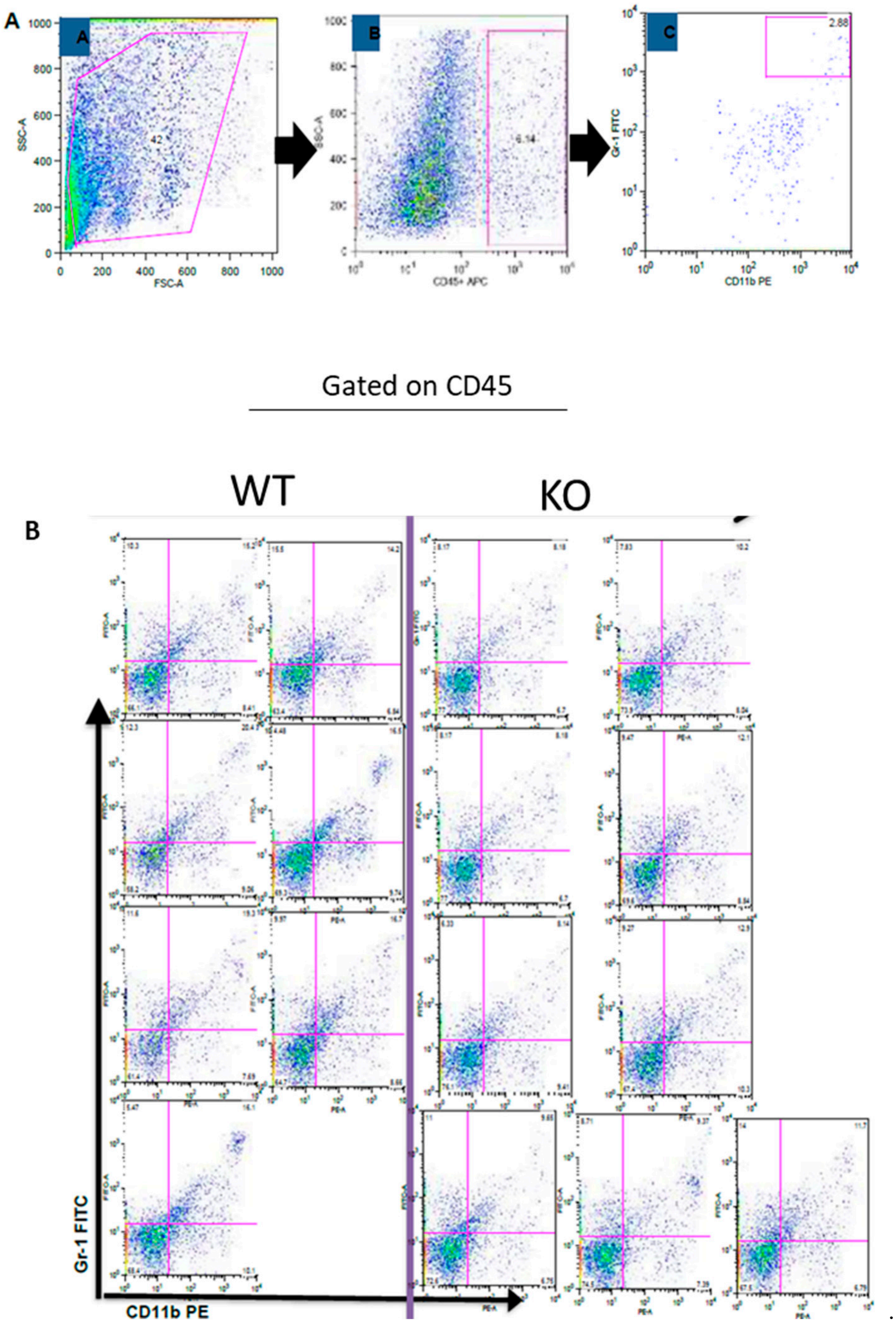

Supplementary Figure S1. CD45+ CD11b+ Gr-1+ cells from wild type and KO derived splenocytes. Representative gating (**A**) and flow cytometry plots of splenic cell suspensions (**B**) analysed for the proportion of CD45+ CD11b+ Gr-1+ cells from properdin wildtype (PropWT) and deficient (PropKO) mice.

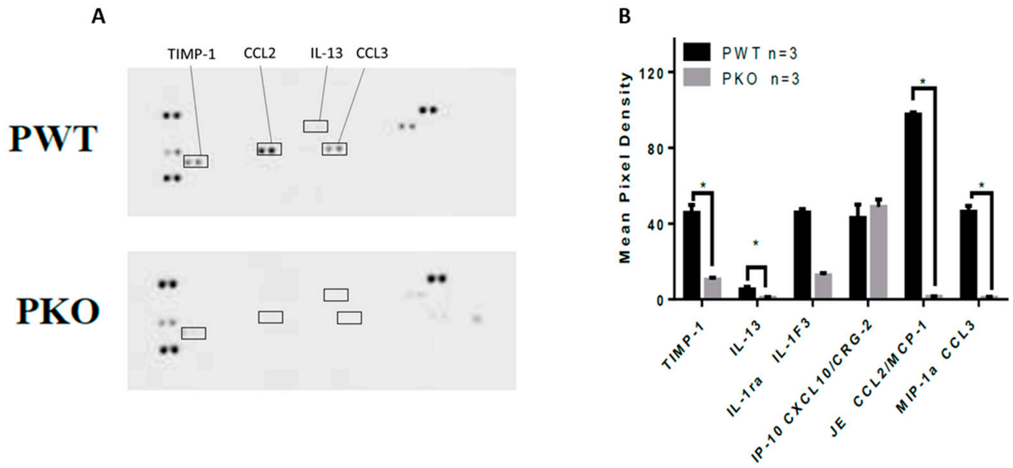

Supplementary Figure S2. Cytokine array analysis. Representative mouse cytokine arrays (**A**) for tumour lysates of wildtype (PropWT) and deficient (PropKO) mice. Three tumours of comparable sizes from each genotype were pooled and extracted protein was analysed using Mouse Cytokine Array (R&D Systems). Reactivities were quantified densitometrically (**B**).
